# Feasibility and Acceptability of Technology-supported Sexual Health Education Among Adolescents Receiving Inpatient Psychiatric Care

**DOI:** 10.1007/s10826-022-02259-4

**Published:** 2022-02-19

**Authors:** Allison E. Olmsted, Christine M. Markham, Ross Shegog, Ana M. Ugueto, Erica L. Johnson, Melissa F. Peskin, Susan T. Emery, Kimberley A. Baker, Elizabeth W. Newlin

**Affiliations:** 1 The University of Texas Health Science Center, 7000 Fannin Street, Houston, TX 77030 USA; 2 1941 East Road, Houston, TX 77054 USA; 3 2800 South MacGregor Way, Houston, TX 77021 USA

**Keywords:** Sexual health, Adolescent health, Online technologies, High risk youth, Preventative intervention

## Abstract

Mental illness in adolescence is associated with high-risk sexual behaviors including multiple sex partners, infrequent or inconsistent condom use, and nonuse of contraception. Inpatient psychiatric care represents a promising setting to provide sexual health education. This pilot study investigates the feasibility and acceptability of online sexual health education in this group by assessing usability and impact on short-term psychosocial outcomes. We administered online modules on healthy relationships, pregnancy prevention, condom use, and sexually transmitted infection (STI) prevention to youth. We evaluated outcomes using a single group, pre/post-intervention design. One quality improvement session assessed staff acceptability of the programming. Participants included 51 inpatients (mean age = 15.3; 61% female; 57% Hispanic or Latino; 55% heterosexual). Overall, the program was feasible to administer and highly acceptable to youth (84-89% liked the modules, 98-100% found them easy to use, 96-100% found them credible, 91-98% said information would lead to healthier dating relationships, and 78-87% would refer to a friend). Youth who completed modules demonstrated improvement in several outcomes: attitudes and norms towards violence (*p* < 0.001), intention to use a method of birth control other than condoms if having sex in the next 3 months (*p* < 0.001), condom knowledge (*p* < 0.001), condom use self-efficacy (*p* < 0.001), condom beliefs (*p* = 0.04), HIV/STI knowledge (*p* < 0.001), and perceived susceptibility to STI (*p* < 0.01). The quality improvement session revealed high acceptability by nursing staff on the unit. This intervention could be useful and efficacious in an inpatient setting and larger studies are warranted to understand its full impact.

## Highlights

- Computer-based sexual health modules were acceptable to inpatient adolescents and feasible to implement.
- Inpatient adolescents improved on knowledge and psychosocial outcomes following online sexual health education.
- Psychiatric units may be viable settings to implement important sexual health education for youth.

Adolescence is a period of increased independence as youth assume greater autonomy from their parents. However, youth often lack the knowledge, resources, and relationship skills to support the development of healthy relationships and avoid unplanned pregnancy or sexually transmitted infections (STI). Youth ages 13 to 24 years account for 21% of all new HIV diagnoses in the United States; ethnic and sexual minority youth represent additional risk populations (Centers for Disease Control and Prevention CDC, 2020). Adolescents account for the largest percentage of undiagnosed cases of HIV (Centers for Disease Control and Prevention CDC, 2020). Half of all new diagnoses of sexually transmitted diseases (STD) occur in young people aged 15–24 (CDC, 2019b). Unplanned pregnancy or STI may negatively impact a youth’s physical and emotional health as well as educational and career goals (Busch et al., 2014). Annual costs to taxpayers for teen pregnancy and STI-related health expenses are estimated at $9 and $6.5 billion, respectively (Chesson et al., 2004; Power to Decide formerly The National Campaign to Prevent Teen and Unplanned Pregnancy, 2013).

In 2020, 9.2 percent of youth (over 2.2 million) ages 12–17 years coped with severe major depression, often co-occurring with other disorders like substance use, anxiety, and disorderly behavior (Reinert et al., 2020). Research supports an association between the presence of mental illness, such as depression and anxiety, and a number of high-risk sexual behaviors in adolescence including multiple sex partners, infrequent or inconsistent condom use, and nonuse of contraception (Brown et al., 2010; Hall et al., 2013; Mazzaferro et al., 2006; Ramrakha et al., 2000). Compared to their healthy peers, youth with a diagnosed psychiatric disorder are 1.2 to 3.9 times more likely to report inconsistent condom use (Brown et al., 2011; Ramrakha et al., 2000). Among adolescents admitted to an acute, inpatient psychiatric unit, those who reported self-cutting were 3.5 times more likely to report infrequent condom use, even after controlling for other factors including sexual abuse history (Brown et al., 2005). This difference is especially pronounced for females with depressive symptoms (Mazzaferro et al., 2006; Rubin et al., 2009; Seth et al., 2011). Similar results have been replicated in longitudinal studies of male and female adolescents (Brown et al., 2006; DiClemente et al., 2001; Lehrer et al., 2006; Seth et al., 2011). Signs of mental illness at baseline also reduce odds of contraceptive use at later time points (DiClemente et al., 2001; Hall et al., 2013; Lehrer et al., 2006).

Lack of contraceptive and condom use among these youth is exacerbated by a number of psychosocial factors, including fear of communication about condoms, perceived barriers to condom use, less perceived relationship control and self-efficacy in negotiating condom use with a new partner, and having norms non-supportive of a healthy sexual relationship (DiClemente et al., 2001; Seth et al., 2011). It is necessary to target these factors to effectively promote healthy sexual behaviors in this population.

Youth with mental illness are more likely to become pregnant and contract an STI. Adolescent females who exhibit psychological distress are more likely to become pregnant than their healthy peers, with odds ranging from 2 times higher at six months follow-up to 2.95 times at five years follow-up (DiClemente et al., 2001; Vigod et al., 2014). Adolescents meeting the criteria for mania were four times more likely to test positive for an STI than youth not meeting the criteria (Brown et al., 2010). Among a birth cohort study of 21-year-olds, those with depressive disorders were 1.6 times more likely than their peers to have a lifetime history of STI (Ramrakha et al., 2000). These data support a focus on adolescents with mental health problems for sexual health interventions.

The 2018 CDC School Health Profile indicated sexual health education in the United States is piecemeal, poorly timed, and information provided is incomplete. Further, the median percentage of schools which teach students about sexual health in grades 6 through 8 has declined by as much as 14% across states since 2008 (CDC, 2019a). Youth also receive sex information of varying quality from multiple other sources such as religious organizations, media, peers, schools, and caregivers (Breuner et al., 2016). Effective interventions to help youth avoid unplanned pregnancy, STI, and related consequences of unhealthy dating relationships are critically needed.

The accumulating evidence that adolescents with mental health concerns are a population at greatest risk of suffering consequences of high-risk sexual behaviors supports mental health professionals taking an active role in addressing sexual health concerns of adolescent patients (Donenberg & Pao, 2005). Given a proportion of adolescents may require hospital admission to address acute psychiatric concerns, inpatient units may represent a promising setting to provide sexual health education. A survey of 100 hospitalized adolescents indicated 37% of females and 44% of males wanted to learn more about contraception and/or abstinence during their stay in the hospital, independent of sexual activity, demonstrating inpatient youth may be open to an intervention of this nature (Guss et al., 2015). In one study on an adolescent psychiatry unit, only 37% of randomly selected youth had sexual health information documented in their charts, suggesting a missed opportunity for these high-risk youth (Harrison et al., 2018). Despite this, there have been few interventions addressing adolescent sexual health in an inpatient setting. However, interventions in similar populations have demonstrated positive results. In a randomized controlled trial (RCT) among adolescents admitted into residential drug treatment programs, youth completing 12 sessions of HIV and STI risk reduction information plus skills-based safer sex training showed improved attitudes towards condoms, reduced frequency of unprotected vaginal sex, and an increased number of adolescents who abstained from sex compared to participants receiving standard care (St Lawrence et al., 2002). Another RCT examined the efficacy of family-based versus adolescent-only HIV prevention programs in decreasing HIV risk and improving parental monitoring and sexual communication among youth receiving inpatient or outpatient mental health treatment. Compared to those in the health intervention, adolescents in HIV prevention interventions reported fewer unsafe sex acts, greater condom use and likelihood of avoiding sex, as well as improved HIV knowledge and self-efficacy (Brown et al., 2014). Finally, Kamke et al. demonstrated online sexual health programming among adolescent girls with emotional and behavioral difficulties improved STI/HIV knowledge, sexual self-efficacy, condom attitudes, and condom norms compared to control participants (Kamke et al., 2020). Despite these successes, findings may not be generalizable to fully inpatient psychiatric youth.

Given the potential missed opportunity of implementing sexual health education during inpatient care, the purpose of this pilot study is to assess the feasibility and acceptability of implementing technology-supported sexual health education to gain input on its relevance and applicability for this population.

## Methods

### Participants

Participants eligible to volunteer were adolescent inpatients (ages 13–17 years) recruited from a large, urban county psychiatric center between September and December 2020. Based on age and diagnosis, the unit admitted *n* = 99 eligible patients during the study period. Of these, clinic staff invited participants using a convenience sample (*n* = 63), based on presence at time of admission as well as if youth would remain on the unit through the days of module administration. After initial contact, staff excluded patients whose primary language was not English (*n* = 5), who carried a diagnosis of moderate to severe intellectual disability (*n* = 1), or who were too psychotic, disorganized, or combative/aggressive to participate in group activities on the inpatient unit (*n* = 2). The final sample included *n* = 51 youth. Due to changes in hospital procedures during the COVID-19 pandemic, we adapted study protocols to adhere to appropriate guidelines outlined by the CDC and our partner unit.

### Procedures

We used a single group pre/post-intervention design. Study staff assessed youth for eligibility at intake and provided admitting parents with information about the study and online sexual health education modules. Parents had the option to decline participation for their child without affecting other care they received. The consent was available in English and Spanish, and included permission for study staff to access their child’s electronic medical records (EMR) to characterize the study population. We obtained youth assent to participate in the study in an individual pre-group meeting. Both the informed consent and assent forms provided information about the sexual health education modules, pre- and post-intervention surveys, types of questions to be asked, voluntary nature of the study, and issues related to confidentiality. If a parent and adolescent both agreed to participate, we enrolled the adolescent into the study. We did not invite youth who did not have parental permission (*n* = 3) to take part in the modules. If a parent consented but the adolescent opted not to participate in the study (*n* = 1), they could still participate in the modules. Declining parents cited that information was unnecessary or could be triggering, and the declining adolescent also stated information was not needed. We matched participant surveys using a unique study ID assigned at pretest. The University Institutional Review Board approved all study procedures.

### Intervention

**Fig. 1:**
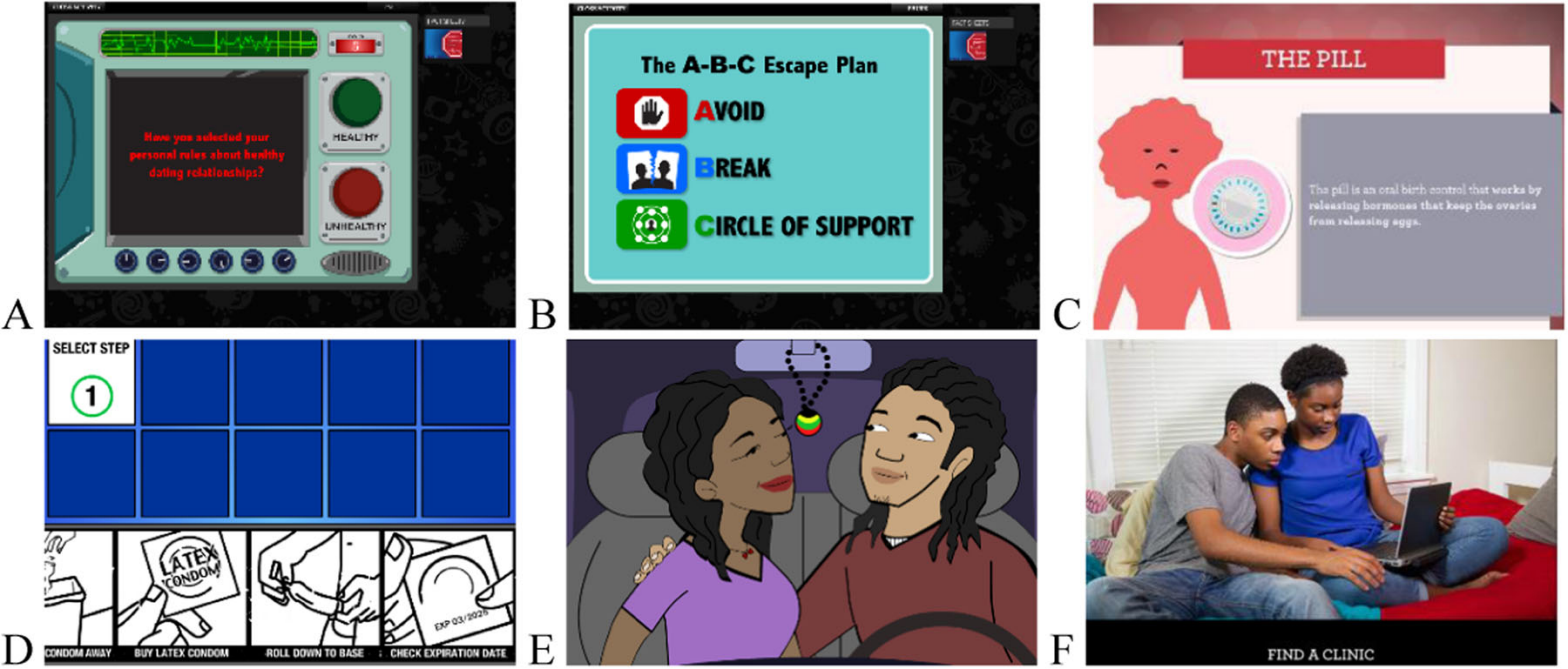
Images from module activities: **A** (*Healthy Relationships*)—Activity in which participants decide if a relationship behavior is healthy or unhealthy; **B** (*Healthy Relationships*) - The **A**–**B**–**C** Escape plan to increase self-efficacy for leaving potentially dangerous situations; **C** (*Pregnancy Prevention*) - Educational session on hormonal contraceptive methods; **D** (*Condom Use*) - Condom use steps interactive activity; **E** (*Condom Use*) - Activity to practice skills negotiating condom use; **F** (*Risk Reduction*) - Real-life story of a couple who must search for a clinic together to be tested

The interprofessional clinical team decided on initial selection and referral to the sexual health education program according to the standard process for the unit. If selected, we offered the program three days a week as part of usual activities for hospitalized adolescents. Nurses surveyed participants individually prior to starting the computer-based sexual health education modules. Study staff delivered the intervention in a quiet room designated for the study and provided headphones for privacy. During each session, youth accessed a preselected set of modules and completed these at their own pace using Wi-Fi on laptop computers. Throughout completion, a study staff member was present to answer any questions or respond to concerns. Four modules were available to complete, each encompassing one of several unique topics: healthy relationships, pregnancy prevention, condom use, and risk reduction (STI/HIV prevention). Each module contained two to six activities related to the topic and took 20–30 minutes to complete. We used activities developed during previous interventions which have demonstrated efficacy among diverse adolescent populations in increasing knowledge and positively changing psychosocial outcomes and/or behaviors related to healthy relationships, pregnancy prevention, condom use, and STI risk reduction (Markham, Tortolero, Peskin et al., 2012; Markham, Peskin, Addy et al., 2017; Markham, Peskin, Shegog et al., 2017; Peskin et al., 2019; Tortolero et al., 2010). Activities were educational and skills-based, providing youth opportunities to apply new knowledge and practice communication through virtual role plays (Fig. 1). We did not tailor modules for this population given the pilot nature of the study.

Youth participated in one to two modules per hour-long session, attending one to two sessions per day depending on time of admission and research staff availability. To ease monitoring of participant attentiveness, research staff preassigned modules by session. Following each module, youth completed the corresponding post-intervention usability feedback survey.

### Measures

#### Participant characteristics

Prior to intervention, we collected demographic measures using a self-administered Qualtrics survey including age, gender, race/ethnicity, country of birth, and sexual identity (Basen-Engquist et al., 1999; The GenIUSS Group, 2013; Tortolero et al., 2010). We extracted data on past medical history from EMR records including primary discharge diagnosis and secondary/comorbid diagnoses based on categories outlined in the fifth edition of the Diagnostic and Statistical Manual of Mental Disorders (DSM-V) (American Psychiatric Association, 2013). We coded risk for suicide as high, moderate, low, or absent (coded as null) based on results from the Columbia Suicide Severity Scale Rating (Posner et al., 2011). We recorded history of abuse or neglect, substance use, prior admission to a psychiatric facility, and suicide attempt as yes/no via medical record reports. We also noted length of stay.

#### Sexual health behaviors

To characterize participants’ sexual experience, the pre-intervention Qualtrics survey also included previously validated items on lifetime and past three-month history of oral, vaginal, and anal sex, including condom use at last sexual intercourse (Ball et al., 2004; Coyle et al., 2001; Tortolero et al., 2010). A subset of these measures reported hormonal contraceptive use at last sex, whereby responses were affirmative (coded as one) if participants indicated using birth control pills, an IUD, a shot (such as Depo-Provera), a patch (such as Ortho Evra), or a birth control ring (such as NuvaRing). We coded hormonal contraceptive use as zero if participants reported using only condoms, “withdrawal or some other method,” or no method at all. We also measured self-reported lifetime history of STI and HIV testing, STI/HIV diagnosis, and pregnancy (Coyle et al., 2001).

#### Psychosocial factors

At pre- and post-intervention, we collected data on psychosocial factors related to sexual behavior and dating relationships. We assessed condom knowledge (α=0.75) and knowledge of HIV/STI (*α* = 0.49) using five items each with yes/no or true/false responses, as well as one question instructing participants to mark signs of an STI (Coyle et al., 2001). We assessed condom beliefs using three items asking about the use of condoms (i.e., “I believe condoms should always be used if a person my age has sex”) with responses assessed on a four-point Likert scale from *strongly disagree* to *strongly agree* (Coyle et al., 2001, *α* = 0.80). We assessed condom self-efficacy using five items measuring how sure the participant was that he or she could do certain behaviors related to condoms (*α* = 0.63). We measured responses on a three-point Likert scale ranging from *not sure at all* to *definitely sure* (Borawski et al., 2005, Coyle et al., 2001). We used two items to measure contraceptive self-efficacy, with a four-point Likert response scale from *I definitely could not* to *I definitely could* (Coyle et al., 2001, Markham, Peskin, Addy et al., 2017, *α* = 0.74). We measured behavioral intentions to use a condom and, separately, to use a method of birth control other than condoms in the next three months using a five-point Likert scale ranging from *not at all likely* to *definitely likely* (Borawski et al., 2005; Markham et al., 2012). We measured perceived susceptibility to STI and HIV using a five-point Likert scale ranging from *no chance* to *a big chance* by asking participants to guess their chances of getting an STI or HIV, respectively, in the next year (Coyle et al., 2001; *α* = 0.55). Finally, we assessed attitudes and norms towards violence using a 10-item scale asking participants to select to what extent they agree or disagree with attitudes towards unhealthy relationship behaviors (i.e., “It is OK for a boy to hit his girlfriend if she insulted him in front of friends”) (α = 0.78). We rated items on a four-point Likert scale ranging from *strongly disagree* to *strongly agree* (Foshee et al., 2001; Orpinas et al., 2013). We calculated Cronbach’s alpha values using baseline data. Values are similar to previous studies, including lower alpha for HIV/STI knowledge which reflects the multidimensional nature of this scale to address specific facts from the intervention (Markham et al., 2012).

#### Usability

Study staff administered paper surveys immediately following each module completion which included usability parameters of likeability, ease of use, acceptability, credibility, perceived impact, and motivational appeal (Markham et al., 2016; Markham, Peskin, Addy et al., 2017; Shegog, Markham, Leonard et al., 2012). We assessed likeability and ease of use using one item each (e.g., “How much did you like these activities?”) on a four-point Likert scale from *like a lot* or *very easy* to *dislike a lot* or *very hard*, with an additional response available to indicate uncertainty. We assessed acceptability using one item asking participants to indicate if activities worked too fast, too slow, or just right. We assessed dichotomous variables of accuracy of information (right/wrong) and trustworthiness (can/can’t be trusted) with the additional option of “don’t know” to improve validity. We assessed perceived impact (i.e., “I think the information I got from these activities will help me have healthier dating relationships”) and motivational appeal (i.e., “Would you tell a friend to try these activities?”) using two dichotomous (yes/no) items with an additional option for uncertain participants. Surveys also included the open-ended question: “What, if anything, would you do to make these activities more useful for people your age?”

### Data Analysis

#### Pre-intervention survey

We conducted descriptive analyses to characterize the sample and assess prevalence of psychosocial variables and behaviors related to dating and sexual relationships. Bivariate analyses between sociodemographic variables and behavior were not significant, and are thus not reported.

#### Usability testing

We analyzed usability factors using descriptive statistics reported by module. In separate analyses, we stratified these results by demographic factors including age, gender, and sexual orientation. We used thematic content analysis to evaluate qualitative responses.

#### Psychosocial outcomes

We used descriptive measures and Wilcoxon Sign-Rank nonparametric tests to analyze changes in psychosocial factors from pre- to post-test for the overall sample. We calculated and reported effect size using the method suggested by Rosenthal when Cohen’s assumptions of normality and homogeneity of variances are violated (1994). Criteria for effect size is low if the value of r varies around 0.1, medium if it varies around 0.3, and large if it varies more than 0.5 (Cohen, 1988). In exploratory, post hoc analyses we sought to understand demographic influences of age, gender, and sexual orientation on psychosocial outcome results. Multivariate analysis was not feasible due to data abnormality; thus, we used Wilcoxon Sign-Rank nonparametric tests for each stratified subgroup.

#### Quality improvement

Following the completion of pilot testing, study personnel hosted one listening session with clinic staff to assess perceptions of the program and suggestions for improvement. The lead author led this group which lasted approximately 45 minutes. We analyzed these results using thematic content analysis.

## Results

### Sample Characteristics

Characteristics of the sample (*n* = 51) are reported in Table 1. The average age for the sample was 15.3 years (range 13 to 17). Of the sample, 31 participants (61%) were female. There was racial diversity, including 29 (57%) Hispanic or Latinos, 8 (16%) Black/African Americans, and 6 (12%) reporting White. The majority (84%) were born in the United States. Over half (55%) reported their sexual identity as straight/heterosexual, and 14 (28%) reported bisexuality. Medical record abstraction revealed the majority of participants (65%) were diagnosed with major depressive disorder, and 67% were at high risk for suicide. Comorbid diagnosis of substance-related and addictive disorder was prevalent at 24%. Prevalence of history of abuse (45%) and substance use (39%) was also high. Nearly one-third (29%) had previously been admitted to a psychiatric facility, and almost half (47%) had a history of suicide attempt. The median length of stay for patients was eight days. Less than half (41%) reported ever having vaginal intercourse, but of these two-thirds (67%) reported vaginal sex in the past three months. Less than one third (29%) reported condom use at last vaginal sex, and only one participant (5%) reported using a hormonal contraceptive method. Three participants (14.3%) reported ever being pregnant or making their partner pregnant. One-third (33%) reported ever having oral sex, and nearly half (47%) of these reported oral sex in the past three months. Only 18% reported condom use at last oral sex. Three participants (5.9%) reported ever having anal sex. Finally, the lifetime history of STI testing was over one-third (35%), while lifetime history of HIV testing was 19%.

**Table 1:** Self-reported characteristics from sample by demographics, medical history, sexual, and testing behaviors (*N* = 51)

	Total Sample (*N* = 51)^a^
Demographic characteristics	*n* (%)
*Age (mean, sd) (years)*	15.3 (1.4)
*Gender*	
Boy/man	16 (31.4%)
Girl/woman	31 (60.8%)
Other	3 (5.9%)
*Race/ethnicity*	
White	6 (11.8%)
Black/African–American	8 (15.7%)
Hispanic or Latino	29 (56.9%)
Other	8 (15.7%)
*Country of birth*	
United States	43 (84.3%)
Other	8 (15.7%)
*Sexual identity*	
Straight/heterosexual	28 (54.9%)
Bisexual	14 (27.5%)
Pansexual	4 (7.8%)
Gay/lesbian	3 (5.9%)
*Medical history*	
Primary diagnosis*	
Depressive disorder	33 (64.7%)
Trauma and stressor-related disorder	9 (17.6%)
Bipolar and related disorders	3 (5.9%)
Other	6 (11.8%)
Comorbid diagnoses*	
Substance-related and addictive disorder	12 (23.5%)
Anxiety disorders	7 (13.7%)
Other	10 (19.6%)
Length of stay (median, range) (days)**	8 (4-26)
High risk for suicide***	34 (66.7%)
*History of abuse or neglect*	23 (45.1%)
*History of substance abuse*	20 (39.2%)
*History of prior admission to psychiatric facility*	15 (29.4%)
*History of suicide attempt*	24 (47.1%)
*Sexual behaviors*	
Ever had vaginal intercourse	21 (41.2%)
Average lifetime vaginal sex partners (sd)	1.9 (1.4)
Vaginal sex in the past 3 months	14 (66.7%)
Condom use at last vaginal sex	6 (28.6%)
Hormonal contraceptive method used at last vaginal sex	1 (4.8%)
Ever been pregnant or made partner pregnant	3 (14.3%)
Ever had oral sex	17 (33.3%)
Average lifetime oral sex partners (sd)	1.6 (0.8)
Oral sex in the past 3 months	8 (47.1%)
Condom use at last oral sex	3 (17.7%)
Ever had anal sex	3 (5.9%)
*STI/HIV testing behaviors*	
Lifetime history of STI test (not including HIV)	9 (34.6%)
Number of times tested for STI (not including HIV) (range)	1 (1-2)
Lifetime history of HIV test	5 (19.2%)
Number of times tested for HIV (range)	1 (1)

*Based on Diagnostic and Statistical Manual of Mental Disorders (DSM-5) diagnostic categories (American Psychiatric Association, 2013)

**Concerns from Child Protective Services can elongate stay, making median a more appropriate measure

***Calculated using scoring from Columbia Suicide Severity Scale Rating (Posner, 2011)

^a^Percentage values not adding to 100% indicate missing values

### Usability Testing

Out of 51 participants, 45 (88%) completed sexual health modules on healthy relationships and condom use, and 46 (90%) completed modules on pregnancy prevention and risk reduction. Out of participants completing modules, 86% completed all four. The primary reason for non-completion was discharge from the hospital. Quantitative usability data for each module is presented in Table 2. Likeability was similar across modules, ranging from 84-89% of participants who reported liking the module “a lot” or “a little”. All modules scored highly on ease of use (reporting modules “very easy” or “kind of easy” to use), ranging from 98–100%. The risk reduction module had the lowest acceptability (“pace of activity just right”) at 87%, though others were all above 90%. Credibility measures (“information correct” and “information trustworthy”) were also high, ranging from 96–100% for each measure, and over 90% (91–98%) of participants reported that the information would help them to have healthier dating relationships. Motivational appeal (“would tell a friend to try this activity”) was lowest across modules, ranging from 78% for condom use to 87% for healthy relationships. Pregnancy prevention and risk reduction modules were each reported at 83%. Analysis of usability by gender demonstrated lower agreement among boys on measures of likeability and motivational appeal for pregnancy prevention and condom use modules. Analysis by age group revealed that motivational appeal for the condom use and risk reduction modules was lower for adolescents ages 16 and 17 years. Other differences when stratified by gender, age group, or sexual orientation were negligible (data not shown).

**Table 2:** Usability ratings based on percent agreement for computer-based sexual health modules by topic

	Module topic			
Parameter (*Gradient*)	Healthy relationships (*n* = 45) *n* (%)	Pregnancy prevention (*n* = 46) *n* (%)	Condom use (*n* = 45) *n* (%)	Risk reduction (STI/HIV prevention) (*n* = 46) *n* (%)
*Likeability* *(“A lot” or “a little”)*	40 (88.9%)	39 (84.8%)	38 (84.4%)	39 (84.7%)
*Ease of use* *(“Very easy” or “kind of easy”)*	45 (100%)	46 (100%)	44 (97.8%)*	46 (100%)
*Acceptability* *(“Pace of activity just right”)*	41 (91.1%)	43 (93.5%)	44 (97.8%)	40 (87.0%)
*Credibility* *(“Information correct”)*	43 (95.6%)	45 (97.8%)	45 (100%)	46 (100%)
*Credibility* *(“Information trustworthy”)*	45 (100%)	46 (100%)	43 (95.6%)	45 (97.8%)
*Perceived Impact* *(“Information will help me to have* *healthier dating relationships”)*	44 (97.8%)	43 (93.5%)	41 (91.1%)	44 (95.7%)
*Motivational appeal* *(“Would tell a friend to try this* *activity”)*	39 (86.7%)	38 (82.6%)	35 (77.8%)*	38 (82.6%)

^*^n = 1 response missing

### Psychosocial Outcomes

Table 3 depicts mean changes in short-term psychosocial outcomes from pre- to post-intervention by module topic for the overall sample. Following participation in the healthy relationships module, participants demonstrated significantly less favorable attitudes and norms towards violence (1.28 vs. 1.18; *p* < 0.001; *r* = − 0.43). Following the pregnancy prevention module, participants reported significantly higher intention to use a method of birth control other than condoms if having sex in the next 3 months (3.40 vs. 4.10; *p* < 0.001; *r* = 0.37). There was a borderline significant increase in contraceptive use self-efficacy (3.20 vs. 3.40; *p* = 0.06; *r* = 0.22). After participating in the condom use module, participants improved in condom knowledge (4.49 vs. 5.78; *p* < 0.001; *r* = 0.59) and condom use self-efficacy (2.38 vs. 2.58; *p* < 0.001 *r* = 0.42). Youth also reported significantly more positive beliefs about condoms (2.38 vs. 2.58; *p* = 0.04; *r* = 0.22). There was no change in intention to use a condom if having sex in the next 3 months (*p* = 0.37). Finally, following participation in the risk reduction module, participants demonstrated significantly higher knowledge of HIV/STI (2.71 vs. 3.57; *p* < 0.001; *r* = 0.42) as well as increased perceived susceptibility to STI (0.34 vs. 0.65; *p* < 0.01; *r* = 0.30). There was no significant change in perceived susceptibility to HIV (*p* = 0.17).

**Table 3:** Short-term psychosocial outcomes pre/post intervention by module topic

	Total sample			
	Pre-test	Post-test	Test statistic	Effect size^b^
	M (SD)	M (SD)	(*p*-value)^a^	*r*
*Healthy relationships* (*n* = 45)				
Attitudes and norms towards violence	1.28 (0.3)	1.18 (0.3)	−4.01 (<0.001)***	−0.43
*Pregnancy prevention* (*n* = 46)				
Contraceptive use self-efficacy	3.20 (0.8)	3.40 (0.7)	1.92 (0.06)	0.22
Intention to use a method of birth control other than condoms if having sex in the next 3 months	3.40 (1.5)	4.10 (1.2)	3.28 (<0.001)***	0.37
*Condom use* (*n* = 45)				
Condom knowledge	4.49 (1.0)	5.78 (0.5)	5.58 (<0.001)***	0.59
Condom beliefs	2.38 (0.5)	2.58 (0.5)	2.01 (0.04)*	0.22
Condom use self-efficacy	2.44 (0.4)	2.66 (0.3)	3.76 (<0.001)***	0.42
Intention to use a condom if having sex in the next 3 months	4.1 (1.3)	4.1 (1.3)	0.89 (0.37)	0.10
*Risk reduction* (*STI/HIV*) (*n* = 46)				
HIV/STI knowledge	2.71 (1.4)	3.57 (1.1)	4.0 (<0.001)***	0.42
Perceived susceptibility to STI	0.34 (0.6)	0.65 (0.9)	2.86 (<0.01)**	0.30
Perceived susceptibility to HIV	0.36 (0.7)	0.54 (0.8)	1.39 (0.17)	0.15

*M* mean, *SD* standard deviation; *p < 0.05, **p < 0.01, ***p < 0.001

^a^Wilcoxon Signed-Rank Non-parametric Test

^b^Effect size calculated using *r* = Z/√*N* where *N* = *n*_1_ + *n*_2_ (Rosenthal, 1994)

Tables 4 through 6 present exploratory, post hoc analyses of psychosocial outcomes stratified by gender, sexual orientation, and age, respectively. When stratifying by gender (Table 4; *n* = 16 boys, *n* = 31 girls), attitudes and norms towards violence and condom knowledge remained significant. Intention to use a method of birth control other than condoms, condom beliefs, condom use self-efficacy, HIV/STI knowledge, and perceived susceptibility remained significant only for girls. Boys showed statistically significant improvements in contraceptive use self-efficacy (2.89 vs. 3.25; *p* < 0.05; *r* = 0.26), and girls showed statistically significant increases in perceived susceptibility to HIV (0.29 vs. 0.48; *p* < 0.05; *r* = 0.35). Stratifying by sexual orientation (Table 5; *n* = 28 heterosexual/straight, *n* = 21 nonheterosexual/straight), attitudes and norms towards violence remained significant. Intention to use a method of birth control other than condoms remained significant only for heterosexual/straight participants (3.11 vs. 3.83; *p* = 0.01; *r* = 0.37). Condom knowledge and condom use self-efficacy remained significant for both groups; however, condom beliefs remained significant only for heterosexual/straight participants, while nonheterosexual/straight participants reported a nonsignificant decline in pre- to post-test mean. Intention to use a condom if having sex in the next three months also decreased nonsignificantly for nonheterosexual/straight participants. Similarly, HIV/STI knowledge remained significant only for heterosexual/straight participants, while nonheterosexual/straight participants reported a nonsignificant decline in the mean score from pre- to post-test. Stratification by age (Table 6; *n* = 27 13–15 years, *n* = 24 16–17 years) revealed that attitudes and norms towards violence remained significant for both groups. Contraceptive use self-efficacy and intention to use a method of birth control other than a condom increased significantly for participants 16–17 years (2.96 vs. 3.39; *p* < 0.01; *r* = 0.43; 3.26 vs. 4.32; *p* < 0.01; *r* = 0.41). Condom knowledge and condom use self-efficacy remained significant for both groups; however, condom beliefs significantly increased only for younger participants. Both age groups demonstrated significant increases in HIV/STI knowledge, though only older participants significantly improved on perceived susceptibility to STI (0.42 vs. 0.91; *p* < 0.01; *r* = 0.42).

**Table 4:** Short-term psychosocial outcomes from intervention by module topic among boys and girls

	Boys (*n* = 16)				Girls (*n* = 31)			
	Pre-test	Post-test	Test Statistic	Effect Size^b^	Pre-test	Post-test	Test statistic	Effect size^b^
	M (SD)	M (SD)	(*p*-value)^a^	*r*	M (SD)	M (SD)	(*p*-value)^a^	*r*
Healthy relationships	1.48 (0.4)	1.28 (0.3)	−2.28 (0.02)*	−0.45	1.17 (0.21)	1.13 (0.24)	−3.06 (0.002)**	−0.41
*Attitudes and norms towards violence*								
*Pregnancy prevention*								
Contraceptive use self-efficacy	2.89 (0.9)	3.25 (0.8)	2.42 (0.03)*	0.49	3.35 (0.6)	3.52 (0.6)	1.14 (0.28)	0.16
Intention to use a method of birth control other than condoms if having sex in the next 3 months	3.19 (1.4)	4.08 (1.1)	1.34 (0.22)	0.22	3.41 (1.5)	4.0 (1.3)	3.05 (0.003)**	0.46
*Condom use*								
Condom knowledge	4.63 (1.0)	5.71 (0.6)	3.06 (0.002)**	0.58	4.45 (1.0)	5.82 (0.5)	4.67 (<0.001)***	0.62
Condom beliefs	2.38 (0.4)	2.56 (0.5)	1.18 (0.28)	0.23	2.34 (0.6)	2.63 (0.4)	2.11 (0.04)*	0.41
Condom use self-efficacy	2.44 (0.4)	2.65 (0.4)	1.78 (0.09)	0.36	2.48 (0.4)	2.66 (0.3)	3.02 (0.002)**	0.43
Intention to use a condom if having sex in the next 3 months	3.81 (1.5)	4.08 (1.3)	0.82 (0.69)	0.16	4.27 (1.1)	4.07 (1.4)	−0.59 (0.56)	−0.08
*Risk reduction (STI/HIV)*								
HIV/STI knowledge	2.75 (1.4)	3.38 (1.4)	1.34 (0.22)	0.26	2.65 (1.5)	3.69 (0.9)	3.67 (<0.001)***	0.48
Perceived susceptibility to STI	0.53 (0.7)	0.85 (1.1)	1.00 (0.63)	0.20	0.26 (0.6)	0.62 (0.9)	2.82 (0.01)*	0.37
Perceived susceptibility to HIV	0.53 (0.6)	0.77 (1.0)	0.45 (0.69)	0.10	0.29 (0.7)	0.48 (0.7)	2.64 (0.02)*	0.35

*M* mean, *SD* standard deviation; *p < 0.05, **p < 0.01, ***p < 0.001

^a^Wilcoxon Signed-Rank Nonparametric Test

^b^Effect size calculated using *r* = Z/√*N* where *N* = *n*_1_ + *n*_2_ (Rosenthal, 1994)

**Table 5:** Short-term psychosocial outcomes from intervention by module topic by sexual identity

	Heterosexual/straight (*n* = 28)				Nonheterosexual/straight (*n* = 21)			
	Pre-test	Post-test	Test statistic	Effect size^b^	Pre-test	Post-test	Test statistic	Effect size^b^
	M (SD)	M (SD)	(*p*-value)^a^	*r*	M (SD)	M (SD)	(*p*-value)^a^	*r*
*Healthy relationships*								
Attitudes and norms towards violence	1.36 (0.4)	1.2 (0.3)	−3.56 (<0.001)***	−0.50	1.2 (0.3)	1.1 (0.2)	−2.4 (0.03)*	−0.41
*Pregnancy prevention*								
Contraceptive use self-efficacy	3.23 (0.9)	3.39 (0.8)	1.73 (0.12)	0.26	3.2 (0.7)	3.4 (0.7)	0.97 (0.35)	0.17
Intention to use a method of birth control other than condoms if having sex in the next 3 months	3.11 (1.5)	3.83 (1.4)	2.50 (0.01)*	0.37	3.89 (1.4)	4.44 (0.7)	1.94 (0.09)	0.37
*Condom use*								
Condom knowledge	4.32 (1.0)	5.73 (0.5)	4.33 (<0.001)***	0.60	4.81 (0.9)	5.94 (0.2)	3.41 (<0.001)***	0.57
Condom beliefs	2.20 (0.5)	2.61 (0.5)	3.01 (0.002)**	0.43	2.58 (0.4)	2.52 (0.5)	−0.81 (0.44)	−0.14
Condom use self-efficacy	2.48 (0.4)	2.64 (0.4)	2.22 (0.02)*	0.33	2.43 (0.4)	2.71 (0.3)	3.09 (0.002)**	0.53
Intention to use a condom if having sex in the next 3 months	3.86 (1.4)	3.96 (1.4)	0.55 (0.79)	0.08	4.55 (0.8)	4.39 (1.1)	−0.78 (0.45)	−0.13
*Risk reduction (STI/HIV)*								
HIV/STI knowledge	2.46 (1.6)	3.58 (1.3)	3.00 (0.002)**	0.43	3.05 (1.1)	3.6 (0.8)	2.56 (0.02)*	0.41
Perceived susceptibility to STI	0.22 (0.5)	0.88 (1.1)	2.98 (0.004)**	0.44	0.52 (0.7)	0.45 (0.7)	−0.52 (1.0)	−0.08
Perceived susceptibility to HIV	0.37 (0.7)	0.63 (0.9)	1.24 (0.22)	0.18	0.38 (0.7)	0.50 (0.7)	0.71 (0.73)	0.11

*M* mean; *SD* standard deviation; *p < 0.05, **p < 0.01, ***p < 0.001

^a^Wilcoxon Signed-Rank Nonparametric Test

^b^Effect size calculated using *r* = Z/√*N* where *N* = *n*_1_ + *n*_2_ (Rosenthal, 1994).

**Table 6:** Short-term psychosocial outcomes from intervention by module topic by age

	13–15 years (*n* = 27)				16-17 years (*n* = 24)			
	Pre-test	Post-test	Test statistic	Effect size^b^	Pre-test	Post-test	Test statistic	Effect size^b^
	M (SD)	M (SD)	(*p*-value)^a^	*r*	M (SD)	M (SD)	(*p*-value)^a^	*r*
*Healthy relationships*								
Attitudes and norms towards violence	1.28 (0.4)	1.18 (0.3)	−3.08 (0.001)**	−0.44	1.29 (0.3)	1.18 (0.3)	−2.55 (0.01)*	−0.40
*Pregnancy prevention*								
Contraceptive use self-efficacy	3.55 (0.4)	3.40 (0.8)	0.62 (0.52)	0.12	2.96 (0.9)	3.39 (0.7)	2.87 (0.004)**	0.43
Intention to use a method ofbirth control other than condoms if having sex in the next 3 months	3.5 (1.7)	3.83 (1.2)	1.93 (0.09)	0.33	3.26 (1.3)	4.32 (1.1)	2.65 (0.009)**	0.41
*Condom use*								
Condom knowledge	4.11 (0.9)	5.68 (0.6)	4.40 (<0.001)***	0.62	4.92 (0.9)	5.90 (0.3)	3.33 (<0.001)***	0.53
Condom beliefs	2.37 (0.6)	2.75 (0.4)	2.78 (0.004)**	0.42	2.39 (0.4)	2.38 (0.5)	−0.04 (0.98)	−0.01
Condom use self-efficacy	2.46 (0.5)	2.64 (0.4)	2.07 (0.04)*	0.31	2.4 (0.4)	2.68 (0.3)	3.35 (<0.001)***	0.56
Intention to use a condom if having sex in the next 3 months	3.96 (1.5)	4.09 (1.4)	0.30 (0.79)	0.04	4.26 (1.1)	4.20 (1.3)	−0.98 (0.43)	−0.16
*Risk reduction (STI/HIV)*								
HIV/STI knowledge	2.30 (1.3)	3.25 (1.1)	3.10 (0.002)**	0.45	3.17 (1.4)	3.91 (1.0)	2.58 (0.01)*	0.39
Perceived susceptibility to STI	0.27 (0.6)	0.42 (0.9)	1.02 (0.50)	0.15	0.42 (0.7)	0.91 (0.9)	2.81 (0.008)**	0.42
Perceived susceptibility to HIV	0.27 (0.7)	0.29 (0.6)	0.06 (1.0)	0.01	0.46 (0.7)	0.82 (0.9)	1.73 (0.12)	0.26

*M* mean; *SD* standard deviation; *p < 0.05, **p < 0.01, ***p < 0.001

^a^Wilcoxon Signed-Rank Nonparametric Test

^b^Effect size calculated using *r* = Z/√*N* where *N* = *n*_1_ + *n*_2_ (Rosenthal, 1994).

### Quality Improvement

Two nurses from the unit participated in a quality improvement session during which they discussed their perceptions of staff, parent, and patient reactions to the program, along with recommendations for improvement. Nurses described positive patient and parent reception of the program, and stated that no one had expressed strong feelings of like or dislike for the programming. They suggested usability improvements for youth including increased privacy during module completion, technology-based education materials for parents, the addition of modules in non-English languages, and a live chat function. Additionally, they stated that module topics on substance abuse and human trafficking would be useful to youth, as well as the incorporation of more case scenarios that increase confidence in getting out of high-risk situations. Module content should be tailored to popular culture to increase youth relatability. From the staff perspective, the modules were very successful. As one nurse stated,

“[The modules help] engage the kids to deal with some issues that are difficult or uncomfortable to talk about…Kids don’t want to read anymore, especially about sex, so they turn to social media and things like that instead. Now we can give them a tech-based resource which is reliable”.

The modules provided much-needed education in a usable format for youth, without requiring additional staff training. They indicated that these activities added substance to prevention programming on the unit.

## Discussion

Youth with mental illness are at higher risk of negative sexual outcomes, but few interventions have been evaluated to reduce this risk. The current study addressed this gap by providing online sexual health modules for inpatient adolescents to investigate feasibility and acceptability of this implementation. Our findings suggest that online sexual health modules are feasible to implement, acceptable to this population, and may improve knowledge and sexual health outcomes for this group.

The sample recruited was diverse with respect to sexuality and relevant behavioral characteristics. The sample included youth with a low prevalence of condom or contraceptive use and a high prevalence of history of depression, attempted suicide, abuse, and substance use, all of which have been linked to increased risky sexual behavior (Brown et al., 2010; Mazzaferro et al., 2006; Ramrakha et al., 2000; Shorey et al., 2015). This suggests that the intervention was well-targeted for this population.

A number of factors support the feasibility for this programming. Most parents and youth reacted favorably when approached for participation, which facilitated recruitment and enrollment into the program. Participants completed each module in 20–30 minutes, allowing flexibility for incorporation into psychiatric unit activities. The median length of stay, eight days, also allowed enough time for programming to reach patients before they left the facility. Staff involvement was not required in the administration of the modules; thus, it did not affect scheduling or other programs in the hospital unit.

Furthermore, youth found the activities to be highly acceptable across usability measures. These results are similar to those found in studies which have expanded and gone on to demonstrate efficacy in randomized controlled trials (Markham et al., 2012; Markham et al., 2014; Peskin et al., 2019; Tortolero et al., 2010). Differences between genders or age groups may have implications for intervention tailoring in future trials, and poorer scores could reflect youth suggestions such as more relatable scenarios, fewer cartoon animations, and more interactive activities. For example, studies on which intervention materials were based used tailoring by gender and sexual experience to target messages about condoms, contraception, and the importance of regular HIV/STI testing for students who were already sexually active (Markham et al., 2012; Peskin et al., 2019). Based on feedback from youth and staff, additional content areas for this population might include substance use prevention, consent, sexual and gender identity, and human trafficking awareness. Activities should be inclusive of sexual and gender minority youth, particularly given their high presence on the unit. Hospital staff remarked on youth’s high acceptability for the online format of the programming; this format allows easy standardization, unlike in-person sessions where comfort and skill level of the educator can affect fidelity (Borawski et al., 2015).

Significant results across several psychosocial outcomes show that this intervention could be efficacious in influencing behavior change among similar groups and corroborate findings from previous studies with similar populations (Brown et al., 2014; Kamke et al., 2020; St Lawrence et al., 2002). Preliminary differences also emerged upon stratification by demographic factors. Although intentions to use contraception in the next 3 months increased significantly overall, contraceptive use self-efficacy increased only among boys. This difference may be influenced by differential social norms around accessing these methods, highlighting the importance of providing access to contraception along with sexual health education to facilitate behavior change. Psychosocial outcomes which remained insignificant in the aggregate or were only significant for girls could indicate that modules were not impactful on these outcomes, or that the sample was underpowered to detect significant effects. Outcomes such as condom beliefs and perceived susceptibility to STI remained significant only for heterosexual/straight participants, while nonheterosexual/straight participants declined on average; this finding could indicate that this participant subpopulation was less likely to see themselves or their needs represented in related modules. Some activities may also be more appropriate for particular age groups, such as the pregnancy prevention module, for which the corresponding outcomes improved only for older participants. The condom use module relied more than others on animation, which could influence the fact that we detected increases only for younger participants, as this may be a more acceptable format for this group. Finally, that perceived susceptibility to STI increased only for older participants could reflect different environmental factors which allowed higher relatability to the on-screen scenarios. Given that we conducted these analyses post hoc, results are preliminary. Larger sample sizes and improved study designs are needed to understand the intervention’s impact on these factors and to more clearly characterize demographic and behavioral trends in this group. However, evidence of initial success supports a larger trial.

This pilot study has several limitations. Small sample size and majority Hispanic/Latino participants limit its generalizability, and the lack of comparison group prevents us from attributing full effects to intervention components. To reduce participant burden, we did not collect additional variables which could be useful to interpretation, such as grade level or household structure. We also assessed post-intervention effects immediately following intervention with no longer-term follow-up, thus limiting ability to assess behavioral changes. Given that this was a pilot study, we did not conduct sample size calculations, and the study may be underpowered to detect effects. However, sample size was consistent with previous usability testing protocols that do not require statistical significance to determine major usability problems (Faulkner, 2003; Nielsen, 1993; Shegog et al., 2012). The structured delivery which required participants to complete back-to-back modules offered that day may have made some youth less likely to engage fully, reducing participation and potential for retention. However, the fact that usability results remained high across modules lends doubt to this influence. Furthermore, structured delivery is more likely to mirror real-life programming given strict inpatient schedules, making this pilot a useful representation of feasibility. Though we did not assess results respective to order of module delivery, this is a potential consideration for future projects.

Future studies should include a control group and increase sample size, ideally through a multisite study to increase external validity, as well as incorporate a longer-term follow-up period to assess impact on behavioral outcomes. Making the intervention available in multiple languages including Spanish could also expand participation. During the COVID-19 global pandemic the average length of stay for patients was longer and patients on the unit were slightly older and more likely to have substance use disorders (Ugueto & Zeni, 2021). More time in the day was also available for modules since the hospital curtailed visiting hours; thus, feasibility would need to be reassessed under normal conditions. However, the fact that the pandemic did not significantly alter the acuity or other demographic factors of patients suggests that effects could remain (Ugueto & Zeni, 2021). The pandemic also made obtaining written consent more difficult as parents and caregivers were only present at the hospital during admission and discharge; thus, there is a possibility for increased recruitment under normal conditions.

These results suggest that inpatient hospital units may be viable settings for adolescent sexual health prevention education. Delivering education by means that youth are accustomed to, including through the use of technology, may allow increased engagement with new information and higher acceptability of programming. Technology-based clinical programming offers ease of standardization, facilitates replication, and reduces initial staff training demands. Sexual health education for adolescents admitted to psychiatric inpatient units provides a unique opportunity that might otherwise be missed to reach a vulnerable youth audience at higher risk of engaging in risky sexual practices. Furthermore, inpatient unit healthcare personnel are readily available to respond to youth who request individual counsel related to their sexual health and similarly available to facilitate discussions with their parent or legal guardian. Thus, interventions tailored to this population may be efficacious in affecting psychosocial factors and behaviors related to risky sexual practices.

We conducted this pilot study during an ongoing global pandemic and, despite initial challenges, successfully recruited participants and implemented the intervention in numbers higher than anticipated. Results showed that these modules were feasible to implement and were largely accepted by participants and supported by staff. Improvement in a number of psychosocial outcomes indicated that sexual health education for inpatient psychiatric youth may reduce risk of negative outcomes. Overall, this pilot study achieved promising results, indicating a larger efficacy study is warranted.

## Data Availability

All data are available at The University of Texas Health Science Center at Houston.
